# Protection of bacteriophage-sensitive *Escherichia coli* by lysogens

**DOI:** 10.1073/pnas.2106005119

**Published:** 2022-03-28

**Authors:** Stanley Brown, Namiko Mitarai, Kim Sneppen

**Affiliations:** ^a^Niels Bohr Institute, University of Copenhagen, DK-2100 Copenhagen, Denmark

**Keywords:** bacteriophage lambda, *Escherichia coli*, herd immunity

## Abstract

Bacteriophage *λ* is a temperate virus infecting the bacterium *Escherichia coli*. Temperate phages have the ability to form lysogens where the prophage is maintained in the infected bacterium in a largely quiescent state. The lysogen becomes immune to superinfection by *λ* by blocking the development of the superinfecting phage. Here we report the *λ* lysogen not only protected itself from killing by *λ* phages but also protected *λ*-sensitive bacteria in mixed culture. This protection required that the lysogen was able to adsorb the superinfecting *λ* phages. The protection was also sensitive to the growth state of the mixed culture, and the *λ* lysogen lost efficiency in protecting *λ*-sensitive bacteria as it stopped growing. A mutant of the *λ* tail protein, *λ*J, was not subject to this loss of protection. Adsorption of *λ* having the wild-type J protein but not the mutant *λ*J protein to *E. coli* was inhibited by interference with bacterial energy metabolism. The last observation suggests wild-type *λ* preferentially infects bacteria with competent energy metabolism.

## Significance

Some viruses that infect bacteria, temperate bacteriophages, can confer immunity to infection by the same virus. Here we report *λ*-immune bacteria could protect *λ*-sensitive bacteria from killing by phage *λ* in mixed culture. The protection depended on the extent to which the immune bacteria were able to adsorb the phage. Reconciling modeling with experiment led to identifying a decline in protection as bacteria stopped growing. Adsorption of *λ* was compromised by inhibition of bacterial energy metabolism, explaining the loss of protection as bacterial growth ceased.

**Fig. 1.**
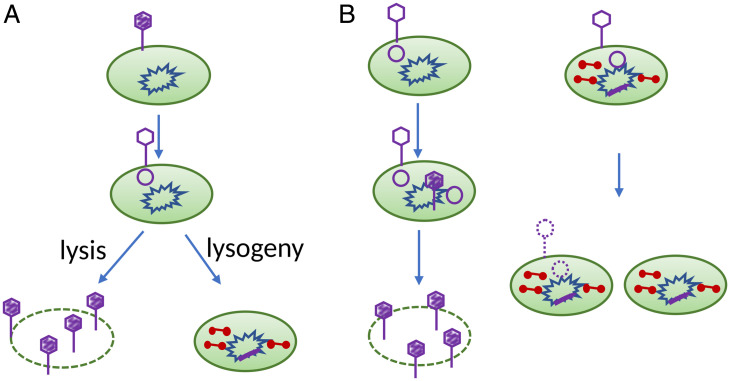
Fates after infection by *λ*. (*A*) On infecting a sensitive *E. coli*, $\lambda^{+}$ (wild-type) can follow one of two developmental pathways. In both cases, the phage chromosome (purple) enters the bacterium and circularizes. If it follows the lytic pathway, it replicates its chromosome, produces the proteins for the viral capsid, assembles the progeny phages, and lyses the infected bacterium, releasing the progeny phages. If it follows the lysogenic pathway, it integrates into a specific location in the bacterial chromosome (blue), and *λ* repressor (red), the product of the *λcI* gene, accumulates, blocking further phage development. (*B*) Pathways followed when *λcI*, *λ* harboring a *cI* mutation, infects nonlysogens (*Left*) or *λ* lysogens (*Right*). (*Left*) When *λcI* infects a nonlysogen, it cannot establish repression and can only enter lytic development. (*Right*) If *λcI* infects a *λ* lysogen, the CI proteins in the cytoplasm block *λ* development. The superinfecting phage is silenced, and its DNA is not replicated but is diluted randomly between the daughter cells during bacterial growth.

Bacteriophage *λ* is a well-studied model system. As a temperate phage, on infecting the bacterium *Escherichia coli*, it can enter one of two developmental pathways (Fig. 1*A* and reviewed in ref. 1). The lytic pathway results in killing the bacterium and releasing on the order of 100 progeny phages. The lysogenic pathway results in the phage integrating its DNA in the bacterial chromosome and producing the *λ*-repressor. The decision of which developmental pathway to follow is made after the *λ* genome enters the bacterial cell and was one of the first molecular switches to be deciphered (2).

The *λ*-repressor, the product of the *λcI* gene, is a DNA-binding protein that blocks RNA synthesis from the lytic promoters of both the resident prophage and superinfecting *λ* phages. In the lysogen, the CI protein also stimulates continued transcription of the *cI* gene. Blocking development of superinfecting *λ* phages is termed immunity (3). The lysogenic *E. coli* grows, and the resident *λ* prophage is stably inherited. *λ* mutants having a defective *cI* gene always enter the lytic pathway on infection of a nonlysogen but are unable to propagate on and kill lysogens (Fig. 1*B* and reviewed in ref. 3). In general, silenced superinfecting *λ* phages do not alter growth rates of lysogens (4). In contrast to lysogens which *λ* can infect but not kill, mutants of *E. coli* that fail to produce the cell-surface protein recognized by *λ*, LamB, are not infected as *λ* does not bind to the bacteria (5, 6).

A different system inhibiting phage propagation, CRISPR, has recently been examined for its ability to protect bacteria against phages in mixed cultures (7). The authors engineered a CRISPR/Cas system so the CRISPR/Cas-harboring bacteria are both unable to propagate the infecting phages and unable to grow if infected. Using this system, they study a fascinating but opposite phenomenon to herd immunity. Instead of measuring how CRISPR/Cas-harboring bacteria protect phage-sensitive bacteria, they measure the ability of a small fraction of sensitive bacteria to propagate a sufficient number of phages to inhibit the growth of the CRISPR/Cas-harboring bacteria. Interestingly, the mathematical treatment they develop is similar to the one we will use below.

Here we examine the protection of sensitive cells by immune cells against phages in mixed culture and how the protection depends on the degree of phage adsorption by the immune cells. In the experiments described below, the *λ* lysogens survived infection unaltered. Since the immune and sensitive bacteria formed colonies of different colors, the survival of each class was monitored independently.

## Results

### Protection of Phage-Sensitive Bacteria.

**Fig. 2.**
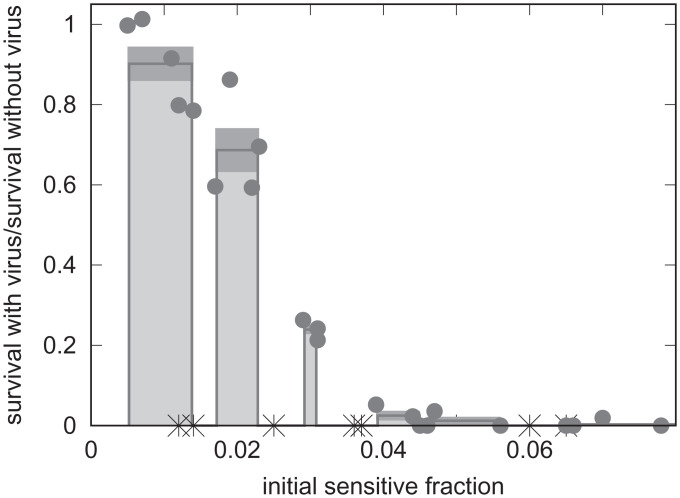
The fractions of surviving *λ*-sensitive bacteria (*y* axis) as function of initial fractions of sensitive bacteria (*x* axis). If infected, *λcIb221* was added to the mixed populations at an MOI of ∼0.003. The cultures without added phage were sampled immediately, diluted, and spread on indicator agar to determine the sensitive fraction of the population (*Materials and Methods*). Cultures with and without added phage were grown overnight before dilutions were spread on indicator agar to determine the surviving sensitive fraction of the populations. The filled circles show the observed fractions of sensitive bacteria (S3137) surviving infection with *λcIb221* in mixed populations with lambda-immune bacteria (S3207). The initial fractions of sensitive bacteria were binned as unit percent ± 0.5 through 5%. Samples with an initial fraction of sensitive bacteria from 6 to 9% were binned as a single group. Within each bin the surviving fraction is shown with the dark band marking the error bars on this average survival. The crosses show data where sensitive bacteria were mixed with bacteria isogenic to S3207 but unable to adsorb *λ* (S3222).

We infected a mixed population of sensitive bacteria, S3137, and immune bacteria, S3207, with *λcIb221* at a low initial multiplicity of infection (MOI = 0.003). We allowed the infected, mixed cultures to grow to saturation. Under these conditions in uninfected mixed cultures, S3137 increased, on average, 930-fold. Our standard conditions are described in *Materials and Methods*. The properties of the bacterial strains that allowed distinguishing them in mixed cultures are summarized in Table 1. The majority of S3137 bacteria survived when S3207 as the immune strain represented 98% or more of the initial mixed population. There was low or no detectable survival of S3137 when S3207 represented 95% or less of the initial mixed population (Fig. 2). The data shown in Fig. 2 with S3207 representing the immune bacteria were a compilation of five independent experiments.

**Table 1.** Properties of recipient bacterial strains after infection with the phages at 37 ${}^{{}^{\circ}}$C

Strain (alias)	Infected with *λcIb221*	Infected with*λ*S3069	Infected with*λcI857*	Colony color
S3137 (sensitive)	Killed	Killed	Killed	Blue
S3207 (immune)	Phage binds, does not kill	amp${}^{\text{R}}$	Phage binds, does not kill	White
S3222 (LamB${}^{-}$)	Phage does not bind	Phage does not bind	Phage does not bind	White

Properties are those of colonies formed at 37 ${}^{{}^{\circ}}$C. Colony color is that on indicator agar. amp${}^{\text{R}}$ indicates resistance to ampicillin.

The observed protection of *λ*-sensitive bacteria was dependent on the ability of the immune bacteria to adsorb the virus. In the experiments shown in Fig. 2, when immune bacteria able to adsorb the virus were replaced with S3222, none of the sensitive bacteria survived. S3222 is isogenic to S3207 but unable to adsorb the virus (*SI Appendix*, Table S5) due to its failure to produce LamB (5, 6). That is, we failed to detect any colonies formed by surviving S3137 (blue on indicator agar) among a total of 2204 colonies formed by S3222 (white on indicator agar). Thus, loss of LamB greatly reduces the ability of the immune hosts to protect the sensitive hosts.

### Measurement of *R*_0_.

**Fig. 3.**
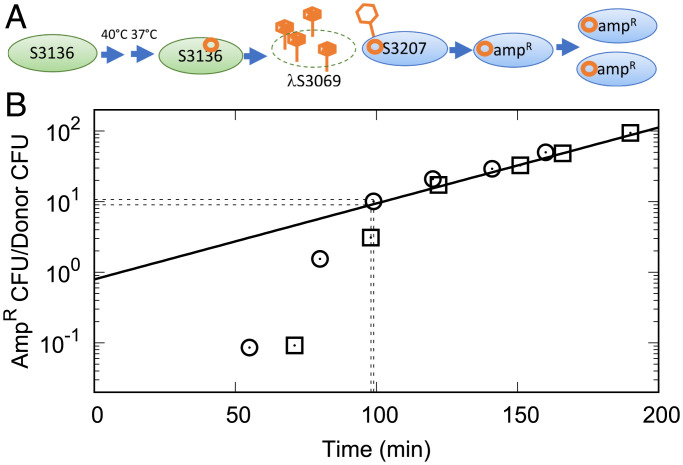
Determination of *R*_0_. (*A*) Schematic description of the measurement. (*B*) The *x* axis is minutes after temperature shift, and the *y* axis is the number of ampicillin-resistant bacteria at 37 ${}^{{}^{\circ}}$C in CFU per donor CFU (30 ${}^{{}^{\circ}}$C ampicillin-resistant CFU prior to temperature shift). Results from experiment 1 are shown as squares and from experiment 2 as circles. The solid line suggests exponential growth of ampicillin-resistant bacteria with a doubling time of 28 min in the later part of experiment 1. Prior to 99 min, the rapid rise of ampicilin-resistant bacteria is assumed to be due to new acquisition of the ampicillin-transducing phage. The dashed lines indicate the extrapolation for the range of initial *R*_0_ values (see *Measurement of R_0_*). Experiment 1 was initiated with 870 CFU/mL S3136 and experiment 2 with 700 CFU/mL S3136.

The protection of sensitive bacteria by immune bacteria bears conceptual similarity to herd immunity in human disease (8, 9). To understand the observed degree of community protection we measured the reproduction number, *R*_0_, or how many bacteria in a fully susceptible population will be infected by viruses released from an infected bacterium. To measure *R*_0_ of *λ* infections directly we used a modified phage, *λ*S3069 (Fig. 3*A*). Since *λ*S3069 had lower infectivity than *λcIb221*, we later corrected for the relative infectivity of the two phages.

Our measurements of *R*_0_ used a primary case lysogen, S3136, that harbored *λ*S3069 as its prophage. *λ*S3069 encodes a temperature-labile *λ*-repressor, CI857 (10, 11). When the temperature is increased, the resident mutant prophage enters lytic development (Fig. 3*A*). *λ*S3069 also carries both a gene for ampicillin-resistance and a ColE1 origin for DNA replication. The ColE1 origin is silent in S3136 because S3136 does not produce DNA polymerase I (12).

Since S3136 does not produce LamB, we expected no loss of released phage due to binding of the primary case host. Thus, following temperature shift, the released phages can only infect the secondary case strain, S3207. In S3207 the endogenous *λ*-repressor blocks viral development and replication from the *λ* origin. However, the ColE1 origin can replicate the circularized infecting phage. Therefore, in S3207, the infecting phage genome, although silenced for phage development, was not diluted through bacterial growth and was able to confer resistance to ampicillin. The *R*_0_ was now measured by counting the number of S3136 colony-forming units (CFU) prior to raising the temperature and the subsequent appearance of transductants of S3207 as ampicillin- and temperature-resistant CFUs (Fig. 3*A* and *Materials and Methods*).

From Fig. 3*B* it is seen that after a time delay of about 60 min from the temperature shift the density of ampicillin-resistant bacteria at 37 ${}^{{}^{\circ}}$C first rose rapidly with an apparent generation time of 6 min. As doubling times for *E. coli* are much longer (13), we interpreted this first, rapidly rising stage as the accumulation of newly transduced S3207. By ∼100 min, the concentration of ampicillin-resistant bacteria began to rise more slowly.

The last time point in the rapidly rising stage of the experiment was taken at 98 min after the temperature shift. A least squares fitting of the later time points of experiment 1 can be described as $690\times 2^{\left(\text{min/28}\right)}$. At 98 min this represents 7,800 CFU/mL (Fig. 3*B*, dashed line); 7,800 CFU/mL secondary case infections divided by the initial 870 30 ${}^{{}^{\circ}}$C ampicillin-resistant primary case CFU/mL gave 9.0 new infections per primary case at this early time point. Repeating the above procedure, the first time point of the slowly rising stage at 99 min of experiment 2 gave 10.7 infections per primary case. Thus, we estimate the early infections to contribute to *R*_0_ by between 9.0 and 10.7 for *λ*S3069.

The increase of ampicillin-resistant colonies after 99 min in Fig. 3 was not only due to the growth of earlier formed transductants. We measured new transductants after the 99 min time point and found they represented additional infections by ∼3.6 per primary case over the next cell generation (*SI Appendix*). Therefore, the resulting total secondary infections per primary case, *R*_0_, of *λ*S3069 rose to between 12.6 and 14.3.

The herd immunity experiments used *λcIb221*, a different phage from *λ*S3069. To convert transduction of ampicillin resistance by *λ*S3069 to infections of *λcIb221* we measured two properties of the phages. First, *λ*S3069 transduced ampicillin resistance at 68% the efficiency it formed plaques (*SI Appendix*, Fig. S2). Second, the burst size for *λ*S3069 was 36% that of *λcI857* as measured by plaque-forming units (PFU) per CFU (*SI Appendix*). The combined corrections raised the total *R*_0_ of 12.6 to 14.3 determined with *λ*S3069 to an *R*_0_ of 52 to 59 for *λcIb221*.

In herd immunity literature, an immune fraction of $1-1/R_{0}$ is often considered to be the herd immunity threshold. Above this threshold, further infections will eventually cease in a population of constant total size (reviewed in ref. 8). However, the latency time of *λ* is about twice the doubling time of sensitive bacteria (*SI Appendix*, Fig. S5); hence, in the experiments summarized in Fig. 2 the phage had only five rounds of infection. Thus, even the maximum *R*_0_ of 59 for *λcIb221* is too small to explain the observed killing of sensitives in Fig. 2.

### Herd Immunity during Exponential Growth.

**Fig. 4.**
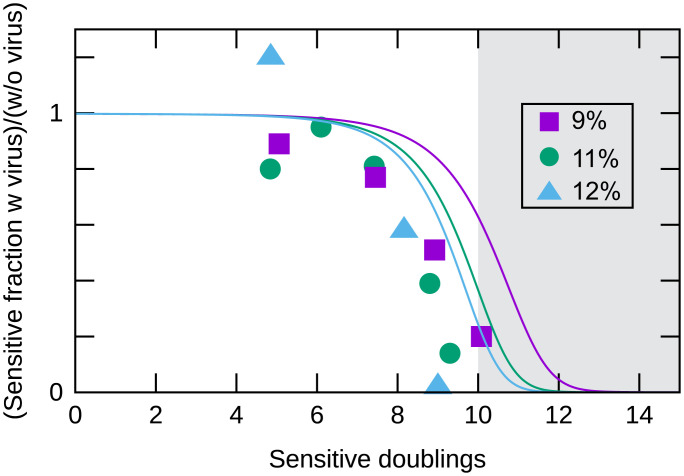
Time course of herd immunity. Mixed cultures of the *λ*-sensitive strain, S3137, and the *λ*-immune strain, S3207, were kept in exponential growth (*Materials and Methods*). The results of three independent experiments are shown. The experiment initiated with S3137 representing 9% of the total CFU is shown with squares, 11% of the total CFU with circles, and 12% of the total CFU with triangles. Model predictions are shown with lines. The average doubling time of S3207 was 28.6 min and of S3137 was 24 min in these experiments. The *x* axis represents generations of S3137 (sensitive strain). The *y* axis represents the fraction of sensitive bacteria surviving with phage divided by the fraction of sensitive bacteria surviving without phage at each time point. Both experiments and simulation were initiated with *λcIb221* added to an MOI of 0.003. The simulation assumed a phage latency time equal to 2.1 doubling times of the sensitive strain and initial bacterial density of $1.4\times 10^{7}$/mL. The line colors represent the initial fraction of sensitive hosts (purple, 9%; green, 11%; cyan, 12%). Growth beyond 10 generations is indicated by a gray area since the cells would enter the stationary phase.

The first herd immunity assay we used allowed the bacteria to grow to stationary phase. We had noticed that at the end of exponential growth, the immune strain, S3207, slowed more rapidly than the sensitive strain, S3137, in mixed cultures (*SI Appendix*, Fig. S4*B*). As our measurements of infection were conducted while the bacteria were in exponential growth, we examined the protection of sensitive bacteria by immune bacteria while both strains remained in exponential growth. The experiment was performed as in our above herd immunity experiments (Fig. 2), but in addition to collecting initial and stationary phase samples we collected samples during growth of the mixed cultures. The results of these experiments are shown in Fig. 4. As expected, with less growth and thus fewer cycles of infection, less killing was observed.

In addition to the corrections to the measurement of *R*_0_ we measured the relative infectivity the two strains to *λ* (*SI Appendix*). In exponential growth, *λ*S3069 generated 1.7 times as many ampicillin-resistant transductants of a *λ*-immune derivative of S3137 as of S3207. Also, as S3137 and S3207 formed colonies of different colors, in each set of experiments we monitored the growth rates of the two strains in mixed, uninfected cultures.

Incorporating the above corrections to the measure of *R*_0_ and the relative infectivity of the two strains, we observed protection of the sensitive strain similar to that predicted by our model (see *Mathematical Modeling of Herd Immunity*) and shown in Fig. 4. Although our measurements and model described the protection of the sensitive strain in exponential growth, we emphasize our observation of approximately threefold greater killing of the sensitive strain when the infected mixed cultures were allowed to enter stationary phase.

### Mathematical Modeling of Herd Immunity.

Our goal is to understand the processes within and between the bacteria that propagate the phages and to describe these processes with mathematical modeling. This goal has guided us to identify features that warranted further investigation. For example, differences in infectivity between the sensitive and immune strains used in Fig. 2 proved critical for our modeling. Also, as the immune strain S3207 had a longer doubling time (*SI Appendix*) than S3137, the ratio of the two strains changed during population growth.

To describe the observed protection of sensitive bacteria and incorporate the growth properties described above, we used the ordinary differential equations that are commonly used to analyze population dynamics in broth culture (14, 15), as well as models applying epidemiology concepts to phage–bacteria interaction (7, 9, 16, 17). Our model assumes the following:

1) The bacteria grow exponentially at constant rates. The sensitive bacteria grow exponentially at a constant rate $g_{S}=\log(2)/24$/min, and the immune lysogens grow exponentially at $g_{L}=\log(2)/28.6$/min (Fig. 4).
2) A free phage particle infects a host cell at a constant rate per phage per sensitive host, *η_S_*, and per immune host, *η_L_* (mL/min). *η_L_* was measured as $6.5\times 10^{-10}$ mL/min (*SI Appendix*, Fig. S6) and *η_S_* ∼ $1.7\times \eta_{L}$ (*SI Appendix*).
3) If a sensitive host is infected by a phage, there is a latency period before cell lysis, and *β* new phage particles are produced. The latency time has average *τ_l_* and follows an Erlang distribution [the interval distribution created by a sequence of steps that happens at a constant rate (18)]. $\tau_{l}=2.1\times \log\;2/g_{S}$ min, and the shape parameter *M* = 8 of the Erlang distribution is obtained by fit to experiment (*SI Appendix*, Fig. S5).

A similar model was employed to analyze infections of mixed populations containing the CRISPR/Cas system (7). The main differences here from their model are that 1) our model employs a latency time distribution fitted to the experimental observation, 2) the sensitive host and the immune host have different growth rates, and 3) the infection rates differ between host types. The detailed model description is given in *SI Appendix*.

We carefully measured the time course of infections and translated this to an effective *R*_0_. Our *R*_0_ primarily reflects the burst size *β*, but since our measurement only takes into account transductants up to a certain time it will not include all released phages. Nevertheless, we found that β≈59, a value consistent with the observed total *R*_0_, gives the best fit to the infection time course data (*SI Appendix*, Fig. S7). This value was used for the primary attempt to fit the herd immunity experiment. In *SI Appendix*, Fig. S8, we simulated how the time course of appearance of newly infected cells depends on the initial host concentration.

The model was applied to the time course of herd immunity in exponentially growing cells (Fig. 4). We see that the discrepancy is quite small, indicating that the model with the measured parameters is consistent with the infection time course in exponential phase.

### Reconciling Results from Exponential Growth and Entry into Stationary Phase.

**Fig. 5.**
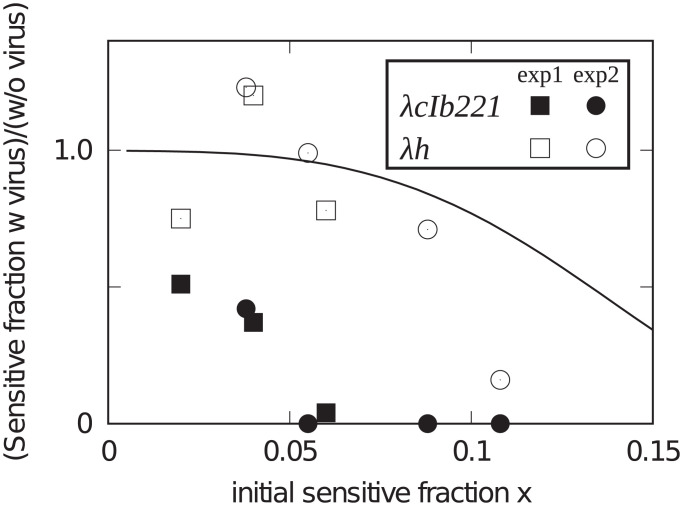
Herd Immunity test with *λcIb221* (filled symbols) and *λh*, *λcIb221J(I1083T)* (open symbols). The solid line is the theoretical prediction using the model and the parameters used for Fig. 4. As in the experiment depicted in Fig. 1, the mixed cultures were allowed to grow to saturation with and without added phages. In these experiments, S3137 increased on average 520-fold in uninfected cultures, thus using about one less host generation time than Fig. 2. Squares are from experiment 1, and circles are from experiment 2. The line represents simulations obtained of survival after nine generations in Fig. 4.

We were able to model the observed herd immunity if the bacteria remained in exponential growth (Fig. 4). However, our model, represented by the line in Fig. 5, failed to describe the observed herd immunity to *λcIb221* if the bacteria were allowed to continue to stationary phase (closed symbols in Fig. 5). That is, protection of sensitive bacteria by immune bacteria declined after departure from exponential growth. A set of observations may suggest a mechanism.

LamB from wild-type *E. coli* freed from the bacterium binds reversibly to $\lambda^{+}$ (19). In mixed cultures the immune strain stopped growing shortly before the sensitive strain (*SI Appendix*, Fig. S4). LamB hyperdiffuses in growing *E. coli* (20), and the hyperdiffusion ceases on energy depletion (21). First, we assumed the absence of hyperdiffusion of LamB on nongrowing *E. coli* would cause it to behave as when freed from the host. As the culture stopped growing, binding to the immune bacteria could become reversible before binding to the sensitive bacteria. An associated shift in the relative adsorption could explain the higher fraction of immune bacteria necessary to protect the sensitive bacteria in outgrown cultures.

We tested the possibility of a biased infection in two ways. First, LamB homologs from other bacteria like *Shigella* (6, 22) and *λ* host range mutants [*λh* (19, 23, 24)] bypass the reversible binding step to purified LamB. To examine the possible involvement of the reversible binding step we isolated a *λh* mutant, *λcIb221 J(I1083T)* (*Materials and Methods*), and repeated the herd immunity experiment allowing the cultures to reach stationary phase. The *λh* mutant, shown as open symbols in Fig. 5, behaved very differently from its parent and closer to the behavior predicted by our simulations.

Second, we asked if phage adsorption was sensitive to energy depletion of the host bacteria. We measured adsorption of *λcIb221* to the immune strain, S3207, in the presence and absence of azide and arsenate, the same inhibitors of metabolism that blocked hyperdiffusion of LamB (21) (*SI Appendix*). The results are summarized in Table 2. For *λcIb221*, removal from a postcellular supernatant, that is, binding to LamB${}^{+}$ bacteria, was sensitive to the inhibition of energy metabolism. This sensitivity was lost with the *λh* mutant and if the LamB${}^{-}$ bacterium, S3222, replaced S3207.

**Table 2.** Energy dependence of adsorption

Experiment (MOI)	Host strain	Ratio unbound *λcIb221*	*λh*
1 (0.002)	S3207	6	0.7
2 (0.015)	S3207	15	2
	S3222	1.2	0.67
3 (0.010)	S3207	11	1.5
	S3222	1.1	0.78

Exponential-phase bacterial cultures were washed and resuspended with buffer. The washed bacteria were preincubated with either added glucose or added sodium azide plus potassium arsenate. Phages were added at the indicated MOI, and incubation continued for 10 min. The adsorption mixes were centrifuged, and the phages remaining in the supernatants were quantified as PFU (25). The concentration of phages in the arsenate plus azide supernatant divided by the concentration in the glucose supernatant is presented. Detailed methods and results are in *SI Appendix*. The *λh* mutant was *λcIb221 J(I1083T)*.

## Discussion

We found herd immunity in our *E. coli*–*λ* system varied continuously with the fraction of immune hosts (Figs. 2 and 5). Our limit of detection was ∼1% survival of sensitive bacteria. Above this threshold of detection, the survival of sensitives increased as the initial fraction of the population immune rose.

Although effective protection of sensitive bacteria from *λcIb221* was provided by immune bacteria, protection was lost if the immune bacteria were replaced with an isogenic strain that failed to produce LamB, the surface protein necessary for phage *λ* infection (Fig. 2). This observation indicates herd immunity, at least in the case of phage *λ*, acts by removing the virus. Although we were unable to find reports experimentally testing the role of adsorption in herd immunity, both a requirement and the lack of a requirement for adsorption have been proposed. A requirement for adsorption has been suggested by Payne et al. (7), and herd immunity conferred by simply diluting the sensitive hosts with insensitive hosts has been proposed by Wang et al. (26).

Interestingly, the protection declined when the bacteria were allowed to enter stationary phase. This was not recapitulated by our mathematical model, pinpointing a qualitative change of phage infection dynamics at this stage. To characterize the nature of this change we replaced *λcIb221* with the host range mutant, *λh*. With *λh*, protection into stationary phase largely behaved as expected from our mathematical model.

We suggest the change after exponential growth could be due to a period of reversibility of $\lambda^{+}$ adsorption that is not observed with *λ* host range mutants (19). That is, the excess killing of sensitive bacteria after departure from exponential growth was due to reversible adsorption to the immune bacteria as the immune bacteria stopped growing before the sensitive bacteria (*SI Appendix*, Fig. S4). Similarly, wild-type *λ* but not *λh* required energy metabolism for adsorption (Table 2).

Although we observed an energy dependence for adsorption and have no evidence for a mechanism, we would like to propose a mechanism and a role for this energy dependence. LamB on the bacterial surface hyperdiffuses in the *E. coli* outer membrane (20) in an energy-dependent manner (21). We envision LamB on the energy-depleted bacteria as analogous to LamB freed from the bacteria in that neither would hyperdiffuse. LamB freed from bacteria causes *λh* mutants but not $\lambda^{+}$ to release their DNA (23, 24). We suggest $\lambda^{+}$ binds to hyperdiffusing LamB in a manner that favors infection more than binding to nonhyperdiffusing LamB. We speculate that this effect may favor phage propagation in natural environments as it allows the phage to diffuse away from energy-depleted bacteria rather than infect a bacterium that may not have sufficient nutrients to complete phage development. The energy dependence of *λ* infection was perhaps observed here because competition studies are conducted in mixed cultures and amplify small differences in growth behavior (27).

In conclusion, we observed community-level protection of sensitive bacteria when mixed with immune bacteria. The possibility of such herd immunity crucially depended on the ability of the immune strain to absorb the infecting phage. Further, we found for phage *λ* that this adsorption decreased with declining energy metabolism of the bacteria. Thus, for wild-type *λ*, *λ*-immune *E. coli* would provide greater community-level protection during growth than when the bacteria enter stationary phase.

## Materials and Methods

### Strains.

Bacterial strains are all derivatives of *E. coli* K12 and are listed in Table 3. Strains S3207 and S3222 are derived from MC4100 (31) with the *λ*–*gal* constellation from ref. 32. We used this defective prophage to provide immunity for two reasons. First, the *rex-gal* deletion removes genes essential for *λ* development and prophage excision preventing the immunity from being transmissible to the sensitive strain. Second, loss of the *λ* left operator, a site removed by the deletion, results in overproduction of CI (33). Strains failing to produce LamB were isolated as spontaneous *λvir^r^* Mal${}^{-}$ mutants as described (5), except maltose utilization was determined with MacConkey agar base supplemented with 0.5% wt/vol maltose.

**Table 3.** Strains

Strain	Description
Bacterial strains	
HO480	F- *polA1(Am) lysA* (28)
S3136	As HO480(*λ*S3069) (11) but *fhuA* and *λvir^r^* Mal${}^{-}$
S3137	F- *lacI^q^ metA endA hsdR17 supE44 thi1 relA1 gyrA96 fhuA*, as S1754 (29) but *fhuA*
S3207	F- *araD139* Δ *(argF-lac)169 flhD5301* Δ *(fruK-yeiR)725(fruA25) relA1 rpsL150 rbsR22* Δ *(fimB-fimE)632(::IS1) deoC1 fhuA (λrex::gfp)* Δ *(rex-galK)::kan*
S3222	As S3207 but *λvir^r^* Mal${}^{-}$
Phage strains	
*λcIb221*	*λcI ET22* Δ*att b221*
*λh*	As *λcIb221* but *J(I1083T*)
*λcI857*	Encodes a thermolabile repressor (10)
*λ*S3069	*λcI857* XbaI::pBluescript KS- (11)
*λvir*	*λ v2, v1v3* (30)

Phage strains are all derivatives of *λ*papa (25). *λ* strains are described in Table 3 and in greater detail in *SI Appendix*.

*λcIb221 J(I1083T)* was isolated as a spontaneous *λh* mutant of *λcIb221* as described (34). The phage still required LamB for infection as it could not form plaques on a strain deleted for *lamB*. Since sequenced *λh* mutants fall between codons 1040 and 1127 (35) of the 1,132-codon *λJ* gene, we sequenced the last 30% of *J* from the *λh* mutant and parent. The sole nucleotide change was in codon 1083.

### Media.

Bacteria were grown in YT broth (36) or on M63 agar (37). Indicator agar was M63 supplemented with 1 µg/mL thiamine, 0.2% glucose, 0.2% decolorized casamino acids, 20 µg/mL L-methionine, 0.2 mM isopropyl β-D-thiogalactoside, and 40 µg/mL 5-bromo-4-chloro-3-indolyl β-D-galactoside. When indicated, sodium ampicillin was added to YT agar to a final concentration of 50 µg/mL. Phages were titered in F-top (36) supplemented with 10 mM MgSO_4_ over a supplemented T-agar (36, 38) (0.8% Tryptone, 0.5% NaCl, 0.1% yeast extract, 5 mM MgSO_4_, 1% agar).

### Infection of Mixed Populations.

We initiated each experiment from an independent single colony by inoculating 2 mL of YT broth and growing with aeration overnight at 30 ${}^{{}^{\circ}}$C. The next day, a saturated culture of S3137, a *λ*-sensitive strain, was diluted 100-fold, and a saturated culture of a *λ* lysogen, strain S3207 or S3222, was diluted 10-fold into YT broth supplemented with 5 mM MgSO_4_. S3207 is *λ*-immune, and *λ* can bind to its surface and inject its DNA. S3222 is identical to S3207 but fails to produce LamB, and *λ* cannot bind to its surface. The diluted cultures were mixed such that each mix received 1 mL diluted S3207 or S3222, 0.1 to 1 mL diluted S3137, and YT 5mM MgSO_4_ to a total volume of 5 mL. The mixed cultures were incubated with aeration at 37 ${}^{{}^{\circ}}$C for ∼40 min. Next, 0.5 mL of the mixed cultures were diluted 10-fold into YT broth supplemented with 5mM MgSO_4_ and with or without 2 × 10^5^ PFU of *λcIb221* for an average MOI of ∼0.003. Samples of the cultures lacking phage were immediately diluted 10^4^-fold into M63 salts, and 100 µL was spread onto indicator agar. On this agar, S3137 forms blue colonies, and both S3207 and S3222 form white colonies (*SI Appendix*, Fig. S1). The plates were incubated at 37 ${}^{{}^{\circ}}$C, and blue and white colonies were counted the next day. The remainder of the broth cultures were incubated with aeration overnight at 37 ${}^{{}^{\circ}}$C. The next day, the saturated cultures were diluted 10^6^ -fold in M63 salts and 50 µL spread onto the above M63-based indicator agar and incubated at 37 ${}^{{}^{\circ}}$C. Blue and white colonies were counted.

Herd immunity during exponential growth was examined similarly except the mixed cultures were sampled during growth, and various dilutions were spread on indicator agar to monitor bacterial growth and survival (Fig. 4).

### Measurement of *R*_0_.

Cultures of the primary case bacterium, S3136, a lysogen harboring *λ*S3069 as a prophage, and the secondary case bacterium, S3207, were grown overnight with aeration in YT broth at 30 ${}^{{}^{\circ}}$C. The next day they were each diluted 10-fold into YT broth supplemented with 5 mM MgSO_4_ and incubated 50 min with aeration at 30 ${}^{{}^{\circ}}$C. To remove any free phages, 1 mL of the diluted S3136 was further diluted into 9 mL M63 salts and the bacteria sedimented at 2,200 × *g*, 5 min at room temperature. The supernatant was decanted and the bacteria resuspended with 10 mL YT 5 mM MgSO_4_ and diluted 1,000 fold with the same. Both 0.1 mL of the washed and diluted S3136 culture and 0.1 mL of the diluted S3207 culture were added to 4.8 mL YT with 5 mM MgSO_4_ for a 10,000-fold excess of S3207. Control cultures of S3136 and S3207 alone were also prepared. A sample of the mixed culture was spread onto YT agar supplemented with 50 µg/mL ampicillin and incubated at 30 ${}^{{}^{\circ}}$C overnight. All cultures were incubated 5 min in a 40 ${}^{{}^{\circ}}$C water bath with shaking and then incubated further at 37 ${}^{{}^{\circ}}$C with aeration. Samples were withdrawn over time and spread on YT agar supplemented with 50 µg/mL ampicillin and incubated at 37 ${}^{{}^{\circ}}$C overnight. Colonies were counted the next day. The 37 ${}^{{}^{\circ}}$C, ampicillin-resistant, Lac${}^{-}$ colonies only appeared with the mixed cultures.

## Supplementary Material

Supplementary File

## Data Availability

All study data are included in the article and/or *SI Appendix*.
