# Supplementary material for: Protection of bacteriophage-sensitive Escherichia coli by lysogens
Source: Proc Natl Acad Sci U S A. 2022 Mar 28;119(14):e2106005119. doi: 10.1073/pnas.2106005119 (PMC9168506; doi:10.1073/pnas.2106005119)
Supplement: Supplementary File [file pnas.2106005119.sapp.pdf]

Supporting Information: Protection of bacteriophage-sensitive *Escherichia coli* by lysogens

Stanley Brown[1], Namiko Mitarai, and Kim Sneppen
Niels Bohr Institute, University of Copenhagen, Copenhagen, Denmark.

Colonies formed by recipient strains

Three recipient strains used in this work, S3137, S3207 and S3208 are shown on the indicator agar. The larger colonies formed by S3137 were expected by the faster growth rate compared with S3207. Colonies formed by the Lac$^-$ strain, S3207, were easily distinguished from the Lac$^+$ strains, S3137 and S3208, by their color on this medium.

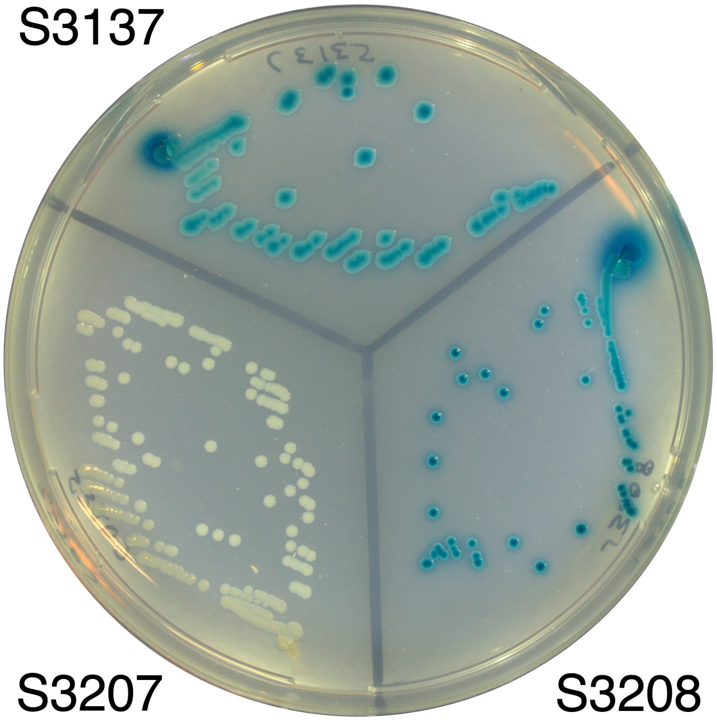

Figure S1: Image of the colonies of the recipient strains (S3137, S3207 and S3208) on indicator agar.

[1]Corresponding author: stanley@nbi.ku.dk

HO480	F- *polA1(Am) lysA* [1]
S2152	F- Δ*(ara-leu)1119* Δ*(argF-lac)169 trp(Am) Mal(Am) fhuA supF*$^+$
S2153	F- *(araD)139* Δ*(argF-lac)169 flhD5301* Δ*(fruK-yeiR)725(fruA25) relA1 rpsL150 rbsR22* Δ*(fimB-fimE)632::IS1* deoC1 fhuA
S3136	as HO480(λS3069) [2] but *fhuA* and λvir^r Mal$^-$
S3137	F- *lacI*^{q} *metA endA hsdR17 supE44 thi1 relA1 gyrA96 fhuA*
S3181	as S2153 but (*argF-lac*)$^+$ *lacI*^{q}
S3207	as S2153 but (λ*rex::gfp)* Δ*(rex-galK)::kan*
S3208	as S3207 but (*argF-lac*)$^+$ *lacI*^{q}
S3222	as S3207 but λvir^r Mal$^-$
S3223	S3137(λ^+)
S3224	S3137(λ*cI857*) λvir^r Mal$^-$

Table S1: Bacterial genotypes: All strains are derivatives of *Escherichia coli K12*. Strains already described in the text are included to ease comparison. S2152 is MBGO*tonA* [3], S2153 is MC4100*tonA* [4] and S3137 is S1754*tonA* [5]. S3207, S3208 and S3222 are derived from S2153 but with the λ-*gal* constellation from [6].

strain (alias)	infected with λ*cIb221*	infected with λS3069	infected with λ*cI857*	colony color
S3137 (sensitive)	killed	killed	killed	blue
S3207 (immune)	phage binds does not kill	amp^R sm^R	phage binds does not kill	white
S3208 (immune)	phage binds does not kill	amp^{R}sm^R	phage binds does not kill	blue
S3222 (LamB$^-$)	phage does not bind	phage does not bind	phage does not bind	white

Table S2: Properties of recipient bacterial strains after infection with the phages at 37°C. Properties are those of colonies formed at 37°C. Colony color is that on indicator agar. amp^R indicates resistance to ampicillin, sm^R indicates resistance to streptomycin.

Expanded description of phage strains

λ*cI857*, Is λ^+ except encoding a thermolabile form of the λ repressor, CI[7]. As a prophage it can be induced to develop by raising the temperature.

λ*cIb221* was a gift from J. Beckwith. It bears the ET22 allele of *cI*, a C to G transversion at nucleotide 37,810 of the λ^+ sequence[8] encoding a defective repressor. It is also deleted for the attachment site used by λ to integrate into the bacterial chromosome. We used this version of a λ*cI* phage to reduce the possibility of stable transfer of the cI^+ allele from the prophage in S3207 to S3137 in the herd immunity experiments.

λh is a host-range mutant described in Materials and Methods. Its complete genotype is λ*cI b221 J(I1083T)*

λ*vir* harbors mutant operators preventing λ repressor (CI) from binding to λ DNA. λ*vir* enters lytic development even when infecting a λ lysogen.

λS3069[2]: λ*cI857* XbaI::pBluescript KS- and encodes ampicillin-resistance

λ*cI A(am)11 nin5* was detected as a spontaneously released phage from S2152(λY18) at 30°C that forms clear plaques at 30°C. λY18 is λ*cI857 A(am)11 nin5* and was a gift of D. Court.

Efficiency of transduction by λS3069

λS3069 lysates were titered on S3137. 800 to 3100 PFU/ml were added to a growing 37°C culture of S3207 at approximately 10^9 CFU/ml in YT broth supplemented with 5 mM $MgSO_4$. The mix was incubated at room temperature for 5 minutes then diluted 5 fold with 37°C YT broth supplemented with 5 mM $MgSO_4$. A sample was removed and spread on YT agar supplemented with 50 μg/ml ampicillin. The rest was incubated at 37°C and samples withdrawn versus time and spread on YT agar supplemented with 50 μg/ml ampicillin. Agar plates were incubated overnight at 37°C and colonies counted the next day. Results from the first experiment are shown in Figure S2. Here, 40 PFU generated 31 ampicillin-resistant transductants for an efficiency of 78%. In two subsequent experiments we observed 167 ampicillin-resistant transductants from 310 PFU and 157 ampicillin-resistant transductants from 220 PFU for efficiencies of transduction of 54% and 71% respectively. The average efficiency of transduction from the 3 experiments was 68%.

The lag in expression of ampicillin-resistance under these conditions was short relative to the generation time of the bacterium as shown in Figure S2.

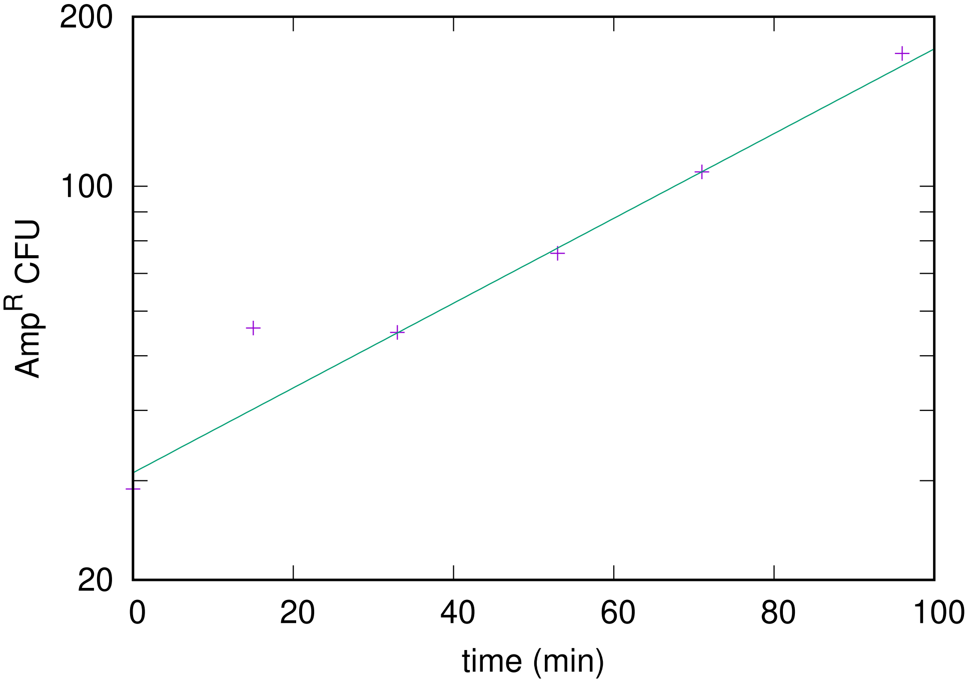

Figure S2: Efficiency of transduction by λS3069: The x-axis represents time of incubation in minutes. The y-axis represents number of ampicillin-resistant colonies observed from spreading 50 μl samples of the mixed culture of S3207 and λS3069. The mixed culture received 40 PFU per 50 μl sample.

Time course of R_0 (delayed R_0)

In our analysis of the initial R_0 (Fig. 3b), we assumed the increase in 37°C ampicillin-resistant CFUs after about 99 minutes was solely due to cell division of transductants formed prior to 98 minutes. A more detailed analysis found new ampicillin-resistant transductants continued to be formed. To observe this, we repeated the temperature-shift experiment with mixed cultures of S3136 and S3207 but at various times diluted the mixed culture into cultures of S3208. Transductants of S3207 and of S3208 were readily distinguished on indicator agar by the color of the colonies (Fig. S1).

We first repeated the assay we used to determine an initial R_0 replacing the rich agar with selective indicator agar which was indicator agar supplemented with 100 μg/ml each, ampicillin and streptomycin. S3136 was streptomycin-sensitive and S3207 was streptomycin-resistant. Here we found an initial R_0 of between 7.8 and 11.5. This value was consistent with our determination of 9.0 - 10.7 on rich agar and indicated we could perform the time course on selective indicator agar.

We next repeated the R_0 measurements but in addition to diluting and spreading the mixed 37°C cultures of S3136 and S3207 on selective indicator agar, we diluted the mixed cultures into a growing culture of S3208. After 10 minutes further incubation with S3208, we spread samples onto selective indicator agar. Throughout the latter period we detected newly formed transductants of S3208 as blue colonies on selective indicator agar at 37°C. We quantified the rate of continued transduction of S3207 by comparing the rate of formation of ampicillin-resistant colonies with the growth rate of the mixed, predominantly S3207, culture as monitored by turbidity. To determine the ratio of S3207 and S3208, samples were withdrawn at time of mixing and optical density at 600nm determined. Unlike in our initial measurements of R_0, the selective indicator agar plates were incubated 2 days at 37°C and transductants of S3207 and S3208 were counted as white and blue colonies, respectively.

The results from one time course experiment are shown in Fig. S3 panels A and B. The mixed culture, predominantly S3207, grew with a doubling time of 29 minutes as determined by increase in absorbance at 600nm. The transductants selected on the agar used in these experiments appeared with an apparent doubling time of 18 minutes from 80 to 120 minutes after temperature shift. The additional growth was probably due to continued transduction as diluting the mixed culture 10 fold into a culture of S3208 generated blue, ampicillin- and streptomycin-resistant transductants over this same period. We are unable to explain the great fluctuations in the appearance of blue transductants and only interpret their appearance to indicate continued active transduction. Also, their continued appearance after the rapid rise of transductants ceased indicates our determination of additional R_0 below may represent a minimum value.

To determine the additional R_0, we assumed transductants appearing by 80 minutes grew with a doubling time of 29 minutes. Thus, excess transductants appearing up to 120 minutes were assumed to be due to new transductions. The 29 minute doubling time predicted 14,300 transductants per ml at 120 minutes but we observed 25,500 transductants per ml for an excess of 11,200 transductants per ml. If each transductant grew with a doubling time of 29 minutes over the 40 minute period and since the sum of $n = 1$ to $n = 40$ of $2^{(n/29)}$ is 68, we expected 11,200/68 or 165 transductants per ml per minute from the 947 "primary case" S3136 CFU/ml. This resulted in a delayed R_0 of 0.174 transductants per ml per min

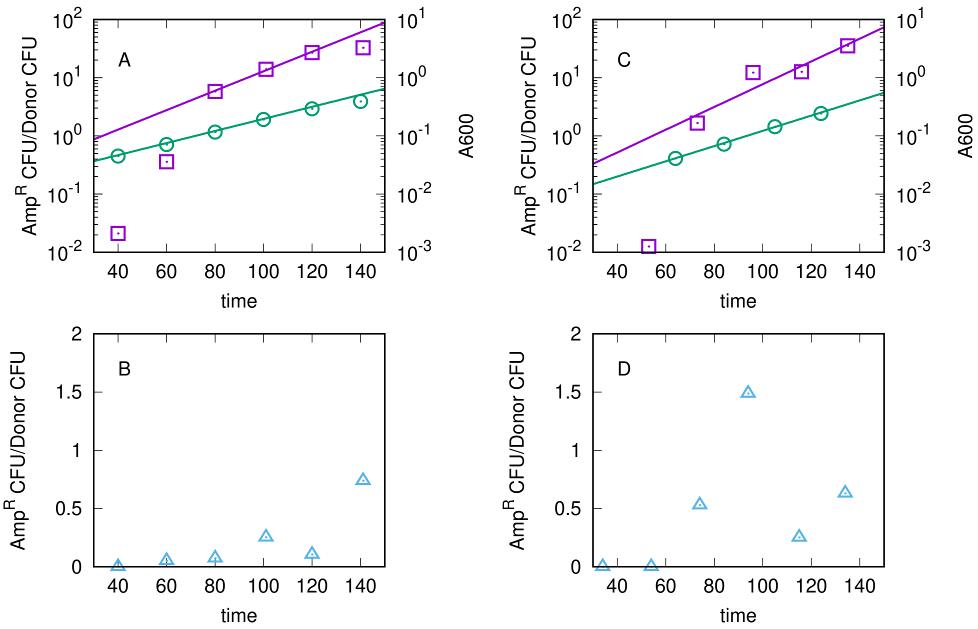

Figure S3: Time course of R_0. In all panels the x-axis is minutes after temperature shift. (AB) The experiment was initiated with 947 CFU/ml of S3136. (A) The y axis is number of ampicillin- and streptomycin-resistant bacteria at 37°C as CFU/ml (squares). The top line in represents a doubling time of 18 minutes. Also plotted is the absorbance at 600 nm of the growing mixed culture (circles, axis shown to the right). The lower line represents a doubling time of 29 minutes. (B) The y axis is number of blue ampicillin- and streptomycin-resistant bacteria at 37°C as CFU/ml 10 minutes after a 10 fold dilution into a culture of S3208. (CD) Repeat of the time course of R_0 experiment. The experiment was initiated with 793 CFU/ml of S3136. (C) The y axis is number of ampicillin- and streptomycin-resistant bacteria at 37°C as CFU/ml (squares). The top line in represents a doubling time of 15.4 minutes. Also plotted is the absorbance at 600 nm of the growing mixed culture (circles, axis shown to the right). The lower line represents a doubling time of 23 minutes. (BD) The y axis is number of blue ampicillin- and streptomycin-resistant bacteria at 37°C as CFU/ml 10 minutes after a 10 fold dilution into a culture of S3208.

per S3136 CFU. We repeated this measurement with independent cultures (Fig. S3 panels C and D) and observed an excess of 6710 transductants per ml from an initial 793 CFU/ml of S3136 or a delayed R_0 of 0.125 transductants per ml per min per S3136 CFU. Under these same growth conditions, S3137, the sensitive strain in the mixed infection experiments grew with a doubling time of approximately 24 minutes and $\lambda cIb221$ had a latency period of between 2 and 3 cell generations (Fig. S4). The additional infections over the period from the second to the third cell generation would be approximately the average of the two above infections per minute times 24 minutes, generating an additional R_0 of approximately 3.6 for transduction of ampicillin-resistance by λS3069.

Burst size corrections

The killing we observed in Figure 2 predicts a much larger value for R_0 than 10.7. To test if this value for R_0 was artificially low due to a difference between the prophage in S3136 and λ*cIb221* we compared three properties of the two systems, burst size, efficiency of transduction of ampicillin-resistance and phenotypic lag of ampicillin-resistance.

To compare burst size we compared S3136 with that of our sensitive host harboring a more wild-type prophage, S3224. S3224 harbors λ*cI857* (see strain descriptions above). YT broth cultures of S3136 or S3224 were established at 30°C from independent colonies. After overnight growth they were diluted 40 fold into YT broth supplemented with 5 mM $MgSO_4$ and grown 1 hour at 30°C. A sample was withdrawn and diluted 100 fold into 4°C M63 salts and the rest temperature-shifted as for the R_0 measurements. When the cultures cleared lysis was completed with a drop of $CHCl_3$ and passed through 0.45 μm sterile filters. Diluted samples of the 30°C cultures were diluted further in M63 salts and spread on YT agar to determine the CFU/ml. The filtrates were diluted with TMG [9] and titered on S3137 to determine PFU/ml. Burst size was determined as PFU/30°C CFU.

In two experiments, the first with 3 independent cultures of each and the second with 2 independent cultures of each, we found λ*cI857* generated burst sizes 2.7 times and 2.9 times the burst size of λS3069, the resident prophage in S3136. The efficiency of packaging of λ phage DNA declines with genome length for phages having larger genomes than wild-type [10]. The DNA of λS3069 is 51,446 base pairs [2] which is 6% longer than wild-type λ DNA and therefore expected to have a smaller burst size.

We measured the efficiency of transduction of ampicillin-resistance of λS3069 phages. In lysates of λS3069 we found the number of transducing units per ml to be approximately 68% the number of PFU per ml. The phenotypic lag for resistance to 50 μg/ml of ampicillin on YT agar represented only a few percent of a generation time (Fig. S2) and was not considered further. Combining corrections for burst size and efficiency of transduction increases the measured initial R_0 of 9.0 - 10.7 for λS3069 by 4.1 fold (2.8/0.68) to 37 - 44 for λ*cIb221*. Applying the same correction factors for the delayed R_0 raised the R_0 for λ*cIb221* by approximately 15 over the next bacterial generation. That is, while 15 secondary infections were generated, the host population doubled.

Departure from exponential growth

This experiment focused on the departure from exponential growth of a mixed culture containing the λ-sensitive strain, S3137, and the λ-immune strain, S3207. The experiment was performed as in the herd immunity experiments except only the uninfected mixed culture was examined. The mixed culture was initiated with 7% S3137 and sampling focused on the period the mixed culture departed exponential growth. S3137 growth as blue CFU/ml is shown as squares and S3207 growth as white CFU/ml is shown as circles. In Fig. S4(a), the lines represent a least squares fit to the data from 0 to 200 minutes. The lower line represents a doubling time of 23.1 minutes and the upper line a doubling time of 28.1 minutes. That is, the doubling time of S3207 was approximately 1.2 fold longer than that of S3137 during the majority of exponential growth. In Fig. S4(b), only the period as growth slowed is illustrated. Here it appears growth of S3207 slowed more rapidly than that of S3137. The lower line represents a doubling time of 31.8 minutes and the upper line a doubling time of 32.2 minutes.

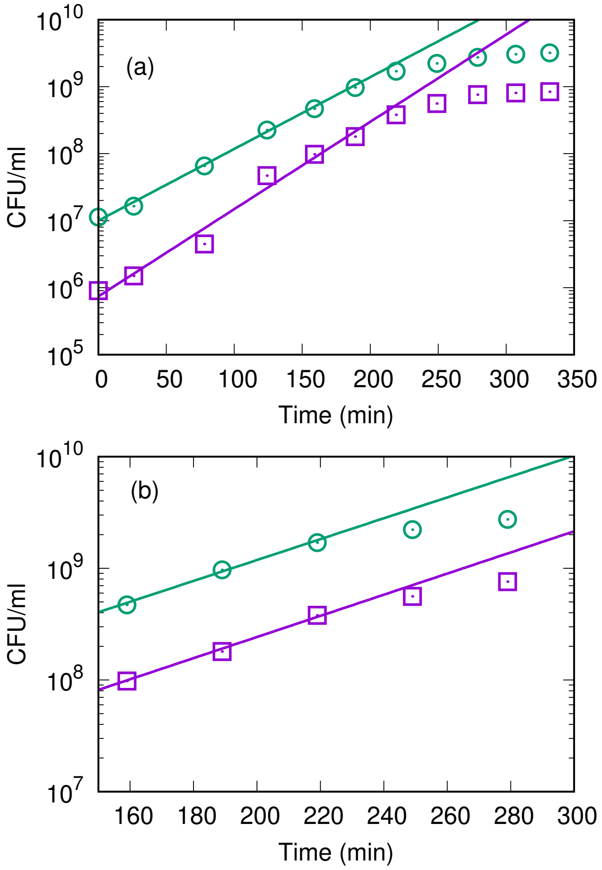

Figure S4: Continued growth of S3137 (squares) compared to S3207 (circles). (a) Growth curve shown from the start of the experiment. The lower line represents a doubling time of 23.1 minutes and the upper line a doubling time of 28.1 minutes. (b) The close-up of the growth curve close to the saturation. The lower line represents a doubling time of 31.8 minutes and the upper line a doubling time of 32.2 minutes.

Infection rate

We measured how the infection rate varied with cell concentration. We measured transduction of ampicillin-resistance into the immune strain, S3207, by λS3069. Overnight cultures of S3207 grown in YT broth 30°C were diluted into YT broth supplemented with 5 mM $MgSO_4$ and incubated at 37°C as in the infection of mixed populations and R_0 experiments. Various dilutions of the culture in YT broth supplemented with 5 mM $MgSO_4$ were mixed with either λS3069 diluted in YT broth supplemented with 5 mM $MgSO_4$ or with YT broth supplemented with 5 mM $MgSO_4$ alone. After 5 and 20 minutes of incubation at 37°C with shaking, samples were withdrawn and diluted 10 fold into 1xM63 salts at 4°C. Samples were spread on YT agar supplemented with 50 μg/ml ampicillin or diluted further in 1xM63 salts and spread on YT agar. Plates were incubated overnight at 37°C and colonies were counted. Phage samples were diluted in TMG and titered on S3137.

Experiment 1

5 min	t0	5 min.	20 min
CFU/ml	PFU/ml	ampR/ml	ampR/ml
6.9 x 10^8	0	0	ND
6.9 x 10^8	4.8 x 10^4	3.84 x 10^4	5.88 x 10^4
2.8 x 10^8	4.8 x 10^4	2.40 x 10^4	4.58 x 10^4
6.9 x 10^7	4.8 x 10^4	7.4 x 10^3	2.0 x 10^4
1.4 x 10^7	4.8 x 10^4	1.6 x 10^3	5.8 x 10^3
0	4.8 x 10^4	0	ND

Experiment 2

5 min	t0	5 min.	20 min
CFU/ml	PFU/ml	ampR/ml	ampR/ml
6.3 x 10^8	0	0	ND
6.3 x 10^8	6.2 x 10^4	4.90 x 10^4	1.01 x 10^5
2.5 x 10^8	6.2 x 10^4	4.00 x 10^4	1.00 x 10^5
6.3 x 10^7	6.2 x 10^4	8.0 x 10^3	3.10 x 10^4
1.3 x 10^7	6.2 x 10^4	1 x 10^3	7.2 x 10^3
0	6.2 x 10^4	0	ND

Table S3: CFU/ml represents initial concentration of S3207 and PFU/ml represents initial concentration of λS3069. ND represents not determined. Values for ampicillin-resistant transductants per ml are the measured values as CFU/ml and not corrected for the observed efficiency of plating of λS3069 transductants.

Relative infectability

We compared the infectability of a derivative of the sensitive strain, S3137, with the immune strain, S3207. S3223 is S3137(λ^+). Since the competitor strain, S3207, is λ-immune, the results would not be confused by λ^+ released from S3223 conferring immunity on S3207. We performed an initial measurement infecting a mixed culture of S3207 and S3223 with the ampicillin-resistance transducing phage, λS3069, to optimize experimental parameters. The preliminary experiment found S3223 acquired ampicillin-resistance approximately 1.8 times as frequently as S3207. We then performed the more careful measurement below using both independent bacterial cultures and independent lysates of λS3069.

Exponential phase cultures in YT broth supplemented with 5 mM $MgSO_4$ were established at 37°C for each strain. They were mixed and diluted into the same medium and grown together for an hour at 37°C. Samples of the mixed cultures were then mixed with either YT broth supplemented with 5 mM $MgSO_4$ or λS3069 diluted into the same. The initial bacterial concentrations were approximately 10^8 CFU/ml. Samples were immediately withdrawn to determine the MOI based on viable cell counts. The samples were then incubated with shaking at 37°C.

After 15 minutes samples were removed and diluted 10 fold into M63 salts at 4°C. Samples were diluted further and spread on indicator agar to determine the ratio of S3137(λ^+) (blue colonies) to S3207 (white colonies). Samples were also spread on indicator agar supplemented with 50 μg/mL ampicillin and incubated at 37°C to examine transductants. The results of the second experiment are shown below. Samples of the mixed bacterial mixed cultures mixed with broth instead of phage or the phage lysates mixed with broth instead of bacteria generated no ampicillin-resistant colonies. Uninfected culture A had a blue/white ratio of 0.586 at 15 minutes. Uninfected culture B had a blue/white ratio of 0.617 at 15 minutes. The average blue/white ratio among ampicillin-resistant transductants was 1.7 times the blue/white ratio in the absence of added phages and ampicillin. That is, S3223 was infected 1.7 times as often as S3207.

Table S4: Relative infectability

mixed bacterial culture	λS3069 lysate	t0 MOI	B/W with amp. divided by without amp
A	A	.0017	1.84
A	B	.0010	1.58
B	A	.0020	1.75
B	B	.0011	1.50

Energy-dependence of adsorption

Herd immunity provided by the λ-immune bacteria declined on departure from exponential growth. To test if adsorption of the phage to the bacteria could cause this departure, we measured adsorption [11] of λ in an energy producing state, in the presence of glucose, and in an energy depleted state, in the absence of glucose but in the presence of potassium arsenate and sodium azide. We performed the experiment 3 times, each bacterial culture was initiated from an independent single colony and each phage lysate was initiated from an independent plaque. Exponentially growing cultures in YT broth were centrifuged (1700 x g, 5 minutes, room temperature) washed with 1/2 volume TMG7.6 (20 mM Tris-HCl pH 7.6, 5 mM $MgCl_2$, 0.1 mg/ml gelatin) and resuspended with 1/4 volume same. 400 μl of resuspended bacteria or TMG7.6 without bacteria were distributed to 2 ml Eppendorf centrifuge tubes. Either 50 μl of TMG7.6 made 2% w/v glucose and 10 mM potassium phosphate pH 7.4 or 50 μl of TMG7.6 made 10 mM in KH_2AsO_4 and 0.2 M NaN_3 were added and the mixtures incubated 10 minutes at 37°C with shaking at 300 RPM. Phage diluted in TMG were added to the indicated MOI. The final concentration of bacteria as determined by counting in a Petroff-Hausser chamber was approximately 3 x 10^9/ml. Incubation with phage was continued for 10 minutes at 37°C with shaking and the bacteria removed by centrifugation at 3,400 x g for 5 minutes at room temperature. 100 μl of the supernatant was aspirated and diluted into 900 μl TMG supplemented with 1 mM potassium phosphate pH 7.4. Further dilutions were prepared in TMG and the phage concentrations determined by spotting 10 μl samples on lawns of S2153. The fraction of phage unbound was the supernatant concentration as PFU/ml in the bacteria-containing mixes divided by the concentration as PFU/ml in the bacteria-free mixes. The results find adsorption by λ*cIb221* is much more sensitive to arsenate and azide than λh and the adsorption requires LamB.

Table S5: Adsorption: Fraction refers to the concentration of phage as PFU/ml in the bacterial supernatant divided by the concentration of phage as PFU/ml in the corresponding buffer alone supernatant. Ratio is the fraction unbound with arsenate and azide (+AsAz) divided by the fraction unbound with glucose and phosphate (+glucose).

Experiment (MOI) Bacteria	phage	fraction +glucose	fraction +AsAz	ratio
1 (MOI 0.002)				
S3207	λ*cIb221*	.003	.022	6
S3207	λh	.001	.0009	.7
2 (MOI 0.015)				
S3207	λ*cIb221*	.0045	.067	15
S3207	λh	.002	.0036	2
S3222	λ*cIb221*	.77	.95	1.2
S3222	λh	.80	.54	.67
3 (MOI 0.01)				
S3207	λ*cIb221*	.0052	.056	11
S3207	λh	.0023	.0036	1.5
S3222	λ*cIb221*	.91	1.0	1.1
S3222	λh	.86	.67	.78

Pilot studies

In our initial efforts to develop the herd immunity assay, we conducted the experiment as shown in Fig. 2 but varied not only the initial fraction sensitive bacteria but also MOI and burst size. To obtain the most accurate survival data in mixed cultures we wanted to be able to quantify both the sensitive and immune strains in the same sample. That is, we wanted to be able to count colonies generated from both on the same plates spread with dilutions of the mixed culture. As described in Fig S1, we did this by using indicator agar where the Lac^+ strain formed blue colonies and the Lac^- strain formed white colonies. As we expected the majority of both the initial and surviving populations to be the immune strain, we used a Lac^+ (blue) sensitive strain and a Lac^- (white) immune strain.

Experiment 1 used λ*cIb221* but a different λ-sensitive strain, S3181, than used for Fig. 2. In experiment 2 we reduced the burst size by replacing λ*cIb221* with λ*cI A(am)11 nin5*. The nonsense allele of A is well suppressed by the tyrosine-inserting suppressor tRNA encoded by *supF* but poorly suppressed by the glutamine-inserting suppressor tRNA encoded by *supE*. Lysates of λ*cI A(am)11 nin5* prepared on the supF strain S2152 form the same number of plaques on S2152 as on S3137 but the plaques are much smaller on S3137 suggesting a smaller burst size on S3137.

Table S6: Experiment 1, λ*cIb221* with S3181 as the sensitive strain and S3207 as the immune strain.

initial fraction sensitive	surviving fraction sensitive MOI=.0004	surviving fraction sensitive MOI=0.004
3%	90%	31%
9%	<3%	<3%
23%	<9%	<9%

Table S7: Experiment 2, λ*cI A(am11) nin5* with S3137 as the sensitive strain and S3207 as the immune strain. The initial MOI was 0.02. Mixed cultures were grown for 9 or 14 cell-generations. Shown is the initial fraction sensitive (blue colonies) for each condition. Phage or broth was added to the mixed cultures to bring the total volume of each to 5 ml. The cultures were incubated at 37°C with aeration for 140 minutes then 0.1 ml samples were diluted into 4 ml of fresh broth. Uninfected cultures were sampled before incubation to determine the initial fraction sensitive. All cultures were sampled after overnight growth at 37°C. The undiluted cultures increased in cell density 430 fold. Shown for the undiluted and diluted cultures is the survival of sensitives (blue colonies) with phage divided by without phage. The greater growth of S3137 than S3207 accounts for recoveries of >100%.

initial	undiluted cultures	diluted cultures
4%	106%	109%
7%	98%	57%
16%	62%	<1%

Theoretical analysis

Continuous time modeling using ordinary differential equations

Virus and host growth are not completely synchronous and the latency time between infection and virus production is distributed. To take into account such a continuous nature of the process, we considered a set of ordinary differential equations which have been used to analyze the well-mixed phage-bacteria system in a broth culture. The main variables include the concentrations of the sensitive hosts, S, the immune hosts, L (lysogens), and the virus, V. Focusing on the exponential phase of growth, we assumed the growth rates of the sensitive hosts, g_S, and and the immune hosts, g_L, are constant. We also assumed that when a sensitive host is infected, it passes through M sequential steps of an infected state before bursting to produce progeny viruses. The number of the steps M were used to adjust the spread of the latency time. The set of equations we used:

$$\frac{dS}{dt} = g_S S - \eta_S \cdot V \cdot S, \tag{1}$$

$$\frac{dL}{dt} = g_L L, \tag{2}$$

$$\frac{dI_1}{dt} = \eta_S \cdot V \cdot S - \frac{M}{\tau_l} I_1, \tag{3}$$

$$\frac{dI_j}{dt} = \frac{M}{\tau_l}\left(I_{j-1} - I_j\right), \quad j = 2, \cdots, M, \tag{4}$$

$$\frac{dV}{dt} = \beta \cdot \frac{M}{\tau_l} \cdot I_M - \eta_s \left(S + \sum_{j=1}^{M} I_j\right) \cdot V - \eta_L \cdot L \cdot V. \tag{5}$$

Here, the average latency time between the infection and the virus production is parameterized by τ_l, and the multiple steps of the infected state control the latency time distribution [12]. The parameters η_S and η_L characterize the virus infection rate to the sensitive hosts and the immune hosts, respectively.

Fitting the latency time distribution using the growth curve of infected S3137

We determined the latency time distribution by monitoring the change in turbidity of a bacterial culture with and without added phages. A culture of S3137 growing at 37°C in YT broth supplemented with 5 mM $MgSO_4$ was diluted into same with or without λ*cIb221* to attain concentrations of 6 x 10^7 bacteria per ml and 6 x 10^8 PFU/ml. The mixes were incubated 5 minutes at room temperature at which λ adsorbs to the bacteria but remains in a lag period before injection of the phage DNA [13]. The mixes were shifted to 37°C to initiate injection and incubated with aeration at 37°C. Versus time, samples were removed and mixed with an equal volume of 1xM63 salts made 300 μg/ml in chloramphenicol and maintained at 2°C. A600 was measured versus YT 5 mM $MgSO_4$ mixed with an equal volume of 1xM63 salts made 300 μg/ml in chloramphenicol. The data is shown in Fig. S5, where time is measured in minutes after shift to 37°C.

The case without phage was fitted by an exponential curve. The data points up to 80 minutes followed a single exponential curve. It was used for the fitting by the least-squares

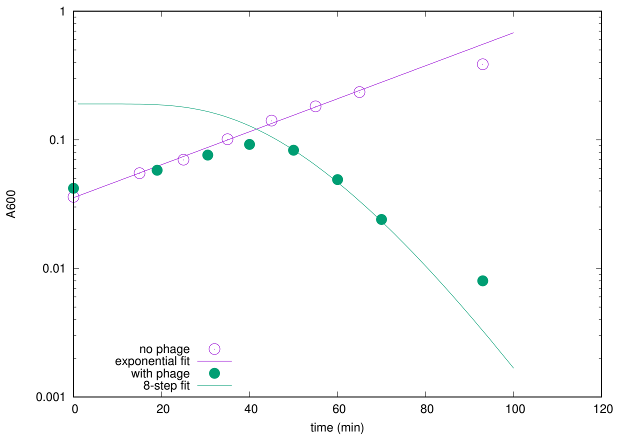

Figure S5: Comparison of the model fitting and the experimentally measured A600 values with (filled circles) or without phage infection (open circles). The last data point that shows A600 below 0.01 is also not fitted, perhaps because such a low adsorption can be due to cell debris. Hence, the fitting was done for the 3 data points between 40 min and 80 min.

Marquardt-Levenberg algorithm provided in gnuplot [14]. This analysis gave the growth rate $g_S = \log(2)/23.5(/\text{min})$ (i.e., the doubling time $\tau_s = 23.5$ min), which is shown as a solid straight line. The case with phage was fitted by the original model (1)-(5) with an initial condition where there are only just infected cells ($S(0) = 0$, $L(0) = 0$, $I_1(0) = 1$, $I_j(0) = 0$ for later latency stages $j = 2, \cdots, M$, $P(0) = 0$). Because the model considers phage production per infected bacterium and does not consider bacterial growth and division early after infection, the initial part of the absorbance simulation of infected bacteria was not fitted. The last data point that shows A600 below 0.01 is also not fitted, perhaps because such a low adsorbance could have been due to cell debris. Hence, the fitting was done for the 3 data points between 40 and 80 minutes. The fitting parameters were the average latency time τ_l, the number of infection step M, and the constant factor C to scale cell density and A_{600}. For a given parameter set, the total cell density at time t, $T(t) = \sum_{j=1}^{M} I_j(t)$, was computed, and the distance from the log scale for the each measurement time point t_k ($k = 1, 2, 3$ represents the 3 data points) was calculated as $\delta = \sqrt{\sum_{k=1}^{3} (\log(C \times T(t)) - \log(A_{600}(t_k))^2}$. We next searched for the parameter set that gave a minimum δ. We found $M = 8$ and $M = 9$ gave a similarly good fit with $\tau_l = 2.1 \times 23.5$ (min) and $\tau_l = 2.2 \times 23.5$(min), respectively. The shown fit in Fig. S5 is $M = 8$ case. In the following, we used the $M = 8$ fit.

Fitting to the infection rate measurement

Next we used a modified model to analyze the infection rate measurement experiment in Table S3. In this experiment, the recipient strain, the strain that was that infected by the phages, was immune. That is, the phages can only infect the initial cells and not propagate. The criterion for survival upon exposure to antibiotics is to have been infected the virus. At time zero, phages are mixed with the recipient strain cells, and the free phage concentration decays as they infect the cells. We used the following set of equations to describe this

situation:

$$\frac{dL}{dt} = g_0 L - \eta_L V L, \quad (6)$$

$$\frac{dR}{dt} = g_0 R + \eta_L V L, \quad (7)$$

$$\frac{dV}{dt} = -\eta_L V (L + R). \quad (8)$$

Here, L is the original recipient that has not been infected, while R is the infected recipient. At time zero, the infected recipient $R(0) = 0$. This model is analytically solvable, for the initial concentration of the recipient $L(0) = L_0$ (CFU/ml) and the phage $V(0) = V_0$ (PFU/ml), the solution is given by

$$L(t) = L_0 e^{gt} \exp\left[-\eta_L V_0 e^{\frac{\eta_L}{g_0} L_0} \frac{1}{g_0} \left(E_1\left(\frac{\eta_L}{g_0} L_0\right) - E_1\left(\frac{\eta_L}{g_0} L_0 e^{g_0 t}\right)\right)\right], \quad (9)$$

$$R(t) = L_0 e^{g_0 t} - L(t), \quad (10)$$

$$V(t) = V_0 \exp\left[\frac{\eta_L}{g_0} L_0 \left(1 - e^{gt}\right)\right], \quad (11)$$

where $E_1(x) = \int_1^\infty \frac{e^{-xt}}{t} dt$ is the exponential integral.

We fitted the solution $R(t)$ to the data in Table S3 to find the best values for the growth rate g_0 and the infection rate η_L. The goodness of the fit was evaluated by minimizing the distance between the measured ampicillin registrant cells per ml at time t_k, ampR(t_k), and the theoretical prediction $R(t_k)$ as $\delta = \sqrt{\sum [\log(0.68R(t_k)) - \log(\text{ampR}(t_k))]^2}$. Here, the factor 0.68 is the efficiency of plating for ampicillin-resistance (see main text). The sum was taken for all initial concentrations, the measurement time points t_k (5 min and 20 min), and the two experimental data sets. Because the CFU/ml at time t_0 in the table was measured for the sample at 5min, the initial condition L_0 was evaluated as that CFU value multiplied by $e^{-g_0 \times 5(min)}$. We have obtained $\eta_L = 6.5 \times 10^{-10}$ ml/min and $g_0 = \log(2)/25$(/min) as the best fit. The comparison between the fitted curve and the experimental data points are showin in Fig. S6. In the following, we set $\eta_L = 6.5 \times 10^{-10}$ ml/min as the infection rate for the immune strain.

Fitting to the R_0 measurement

Next we used the slightly modified full model to simulate the R_0 measurement experiment. Instead of the sensitive strain, we have a donor strain that at time zero is set to the "infected" state 1. The recipient will be infected by the phage but they are immune. The criterion for survival upon exposure to antibiotics is to have been infected the virus, as in the infection rate measurement. The donor did not absorb the phages. Therefore, we use the following

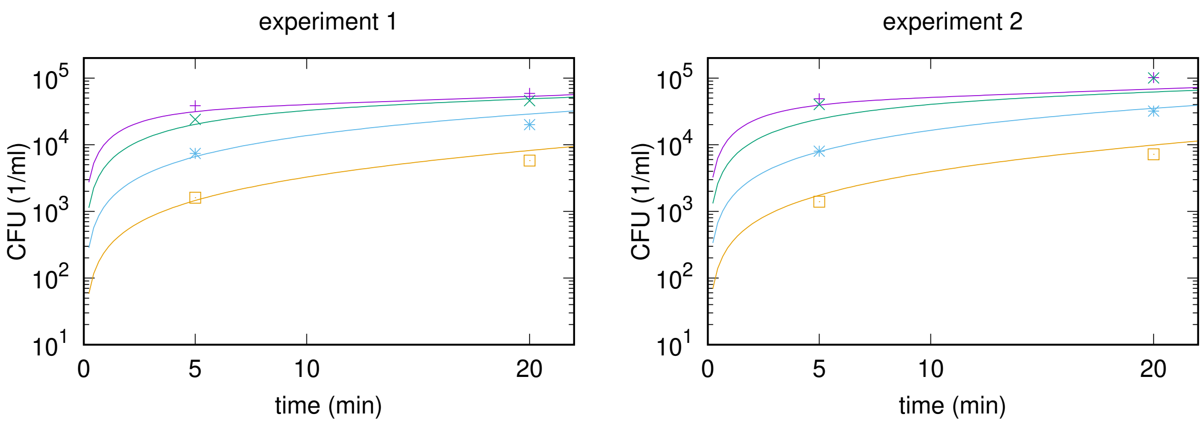

Figure S6: Comparison between the infection rate experiment data (points, from Table S3) and the fitted curve (lines). The color indicates the correspondence between the data and the fit. The initial bacteria density was 1.1×10^7 CFU/ml, 3.4×10^7 CFU/ml, 9.0×10^7 CFU/ml and 22×10^7 from bottom to top respectively into S3137. Infecting phage density was always 1.0×10^5 PFU/ml.

model to simulate the R_0 measurement:

$$\frac{dL}{dt} = g_0 L - \eta_L V L, \tag{12}$$

$$\frac{dR}{dt} = g_0 R + \eta_L V L, \tag{13}$$

$$\frac{dI_1}{dt} = \begin{cases} 0, & \text{if } t < t_w \\ -\frac{M}{\tau_l} I_1, & \text{if } t \geq t_w \end{cases} \tag{14}$$

$$\frac{dI_j}{dt} = \frac{M}{\tau_l}\left(I_{j-1} - I_j\right), \quad j = 2, \cdots, M, \tag{15}$$

$$\frac{dV}{dt} = \beta \cdot \frac{M}{\tau_l} \cdot I_M - \eta_L V(L + R). \tag{16}$$

Here, L represents the original recipient that has not been infected, and R the infected recipient. We have two independent experimental data sets, and fitting was done for each data set separately. First, the growth rate g_0 was estimated by fitting to the exponential function to the OD curve by the least-squares Marquardt-Levenberg algorithm provided by gnuplot. We have obtained $g_0 = log(2)/31$ (1/min) and $g_0 = log(2)/24$ (1/min) for experiment 1 and 2, respectively (Fig. S7).

We then use $M = 8$ and $\tau_l = 2.1 \times \log(2)/g_0$, as obtained by the fitting to the latency time distribution of infected S3137. Because the R_0 measurement was done by prophage induction and induction requires additional time for the prophage to be ready to reproduce, we added this additional time t_w as a fitting parameter. In the model, t_w is the time between the temperature shift and the start of the lysis process. The distributed production of infected cells R comes from this delay time plus the latency time distribution.

The initial conditions used for measuring the delayed R_0 (Fig. S3) were used to simulate the observed results. That is, the initial conditions for experiment 1 used $L(0) = 1.0 \times 10^7$ (/ml), $I_1(0) = 947$ (/ml) and for experiment 2 used $L(0) = 3.8 \times 10^6$ (/ml) and $I_1(0) = 793$ (/ml). The infection rate determined in the previous section, $\eta_L =$

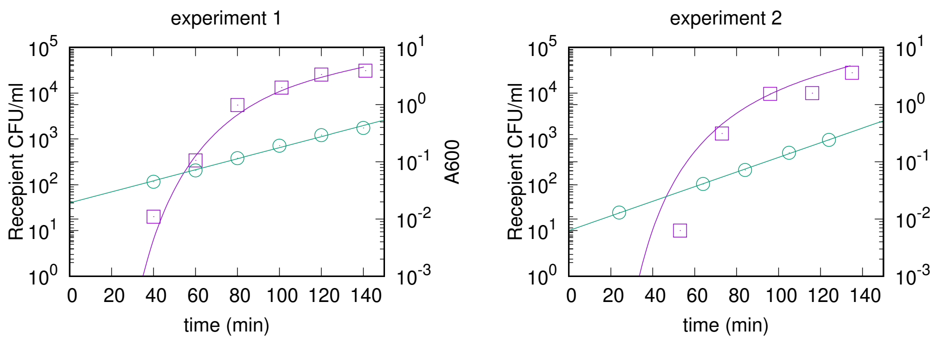

Figure S7: Comparison of time course of newly infected population corrected for the efficiency of transduction $0.68 \times R(t)$ and the data. Green: No phage, turbidity. Purple: ampicillin-resistant CFU/ml. Circles are squares represent acquired data. The lines represent fits from the model.

6.5×10^{-10} ml/min, was used. We varied the burst size of the individual event β and the waiting time t_w and searched for the parameter set that minimized the deviation $\delta = \sqrt{\sum \left((\log(0.68R(t_k)) - \log Amp^i(t_k)) \sqrt{N_k^i} \right)^2}$ from the infected recipient concentration. Here, $Amp^i(t_k)$ is the ampicillin resistant CFU/ml for experiment i at time point t_k, and N_k^i is CFU counted on the plate to take into account the error due to limited sampling. The factor 0.68 is the efficiency of plating for ampicillin-resistance (see main text). As the best fit, we have obtained the delay time $t_w = 17$ min and the bust size per donor $\beta = 21$. The comparison between the experimental data and the fit is shown in Fig. S7.

Using the fitted parameters, we have simulated how the "R_0" defined as the number of newly infected recipient cells per donor cell changes over time. We set the burst size to be $\beta = 21 \times 2.8 = 58.8$, where the factor 2.8 is from the burst size correction between the donor strain in the R_0 measurement and the sensitive strain used in this experiment (see main text). The result is shown in Fig. S8 left. Here, we have used the growth rate to be $g_0 = log(2)/30\text{min}$, and We set $t_w = 0$, i.e., donors are assumed to be just infected at time zero and immediately move to the phage production process. We set the initial amount of the donor cells to be $I_1(0) = 10^4$/ml. We see the low recipient density gives a significant delay in the rise of "R_0", while for high initial density "R_0" quickly saturates to the burst size $\beta \approx 59$. To visualize this initial density dependence clearer, we also show "R_0" at the time point $2\tau_l$, the time scale at which the next round of burst would start if the newly infected cells were sensitive, as a function of initial recipient density (Fig. S8 right).

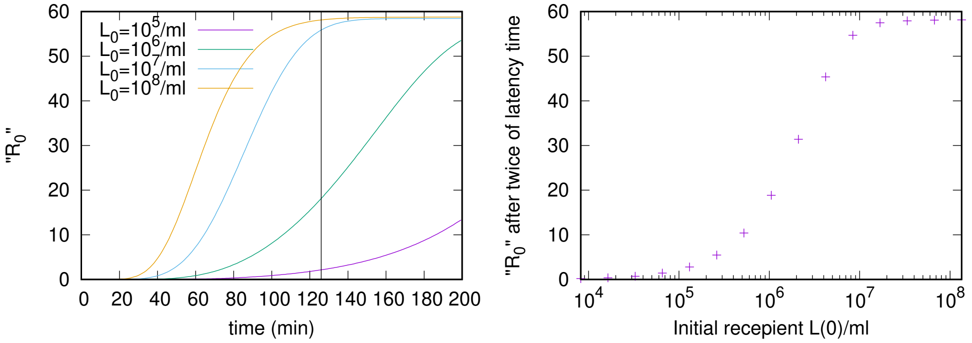

Figure S8: Left: Simulated newly infected recipient cells per donor cell as a function of time for various initial concentration of recipient cell L_0. The vertical black line indicates the time point $2\tau_l$, the time scale at which the next round of burst would start if the newly infected cells were sensitive.

References

[1] Hove-Jensen B (2008) Two-step method for curing *Escherichia coli* of ColE1-derived plsmids. *Journal of Microbiological Methods* 72(2):208–213.

[2] Marchi AN, Saaem I, Vogen BN, Brown S, LaBean TH (2014) Toward larger DNA origami. *Nano Letters* 14(10):5740–5747.

[3] Brown S, Brickman E, Beckwith J (1981) Blue ghosts: a new method for isolating amber mutants defective in essential genes of *Escherichia coli*. *Journal of Bacteriology* 146(1):422–425.

[4] Casadaban MJ (1976) Transposition and fusion of the *lac* genes to selected promoters in *Escherichia coli* using bacteriophage λ and mu. *Journal of Molecular Biology* 104(3):541–555.

[5] Thon G, Bjerling KP, Nielsen IS (1999) Localization and properties of a silencing element near the *mat3-M* mating-type cassette of *Schizosaccharomyces pombe*. *Genetics* 151(3):945–963.

[6] Baek K, Svenningsen S, Eisen H, Sneppen K, Brown S (2003) Single-cell analysis of λ immunity regulation. *Journal of Molecular Biology* 334(3):363–372.

[7] Sussman R, Jacob F (1962) On a thermosensitive repression system in the *Escherichia coli* λ bacteriophage (Article in French). *C. R. Acad. Sci. (Paris)* 254:1517—1519.

[8] Daniels D, Schroeder J, Szybalski W, Sanger F, Blattner F (1983) Appendix i a molecular map of coliphage lambda. *Lambda II* Monograph 13:469–517.

[9] Silhavy TJ, Berman ML, Enquist LW (1984) *Experiments with Gene Fusions.* (Cold Spring Harbor Laboratory).

[10] Weil J, Cunningham R, Martin R, Mitchell E, B. B (1972) Characteristics of λp4, a λ derivative containing 9% excess DNA. *Virology* 50:373–380.

[11] Hendrix RW, Duda RL (1992) Bacteriophage λ PaPa: not the mother of all λ phages. *Science* 258(5085):1145–1148.

[12] Mitarai N, Brown S, Sneppen K (2016) Population dynamics of phage and bacteria in spatially structured habitats using phage λ and *Escherichia coli*. *Journal of Bacteriology* 198(12):1783–1793.

[13] Mackay D, Bode V (1976) Events in lambda injection between phage adsorption and DNA entry. *Virology* 72:154–166.

[14] Williams T, Kelley C, many others (2010) Gnuplot 4.4: an interactive plotting program (`http://gnuplot.sourceforge.net/`).
